# Reservoir computing using dynamic memristors for temporal information processing

**DOI:** 10.1038/s41467-017-02337-y

**Published:** 2017-12-19

**Authors:** Chao Du, Fuxi Cai, Mohammed A. Zidan, Wen Ma, Seung Hwan Lee, Wei D. Lu

**Affiliations:** Department of Electrical Engineering and Computer Science, University of Michigan, Ann Arbor, MI 48109 USA

## Abstract

Reservoir computing systems utilize dynamic reservoirs having short-term memory to project features from the temporal inputs into a high-dimensional feature space. A readout function layer can then effectively analyze the projected features for tasks, such as classification and time-series analysis. The system can efficiently compute complex and temporal data with low-training cost, since only the readout function needs to be trained. Here we experimentally implement a reservoir computing system using a dynamic memristor array. We show that the internal ionic dynamic processes of memristors allow the memristor-based reservoir to directly process information in the temporal domain, and demonstrate that even a small hardware system with only 88 memristors can already be used for tasks, such as handwritten digit recognition. The system is also used to experimentally solve a second-order nonlinear task, and can successfully predict the expected output without knowing the form of the original dynamic transfer function.

## Abstract (web summary)

Reservoir computing facilitates the projection of temporal input signals onto a high-dimensional feature space via a dynamic system, known as the reservoir. Du et al. realise this concept using metal-oxide-based memristors with short-term memory to perform digit recognition tasks and solve non-linear problems.

## Introduction

**Fig. 1:**
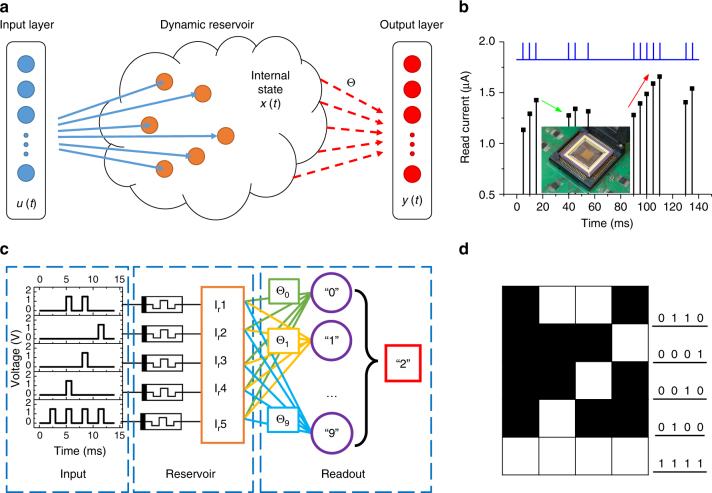
Reservoir computing system based on a memristor array. **a** Schematic of an RC system, showing the reservoir with internal dynamics and a readout function. Only the weight matrix Θ connecting the reservoir state *x(t)* and the output *y(t)* needs to be trained. **b** Response of a typical WO_*x*_ memristor to a pulse stream with different time intervals between pulses. Inset: image of the memristor array wired-bonded to a chip carrier and mounted on a test board. **c** Schematic of the RC system with pulse streams as the inputs, the memristor reservoir and a readout network. For the simple digit recognition task of 5 × 4 images, the reservoir consists of 5 memristors. **d** An example of digit 2 used in the simple digit analysis

Reservoir computing (RC) is a neural network-based computing paradigm that allows effective processing of time varying inputs^1–3^. An RC system is conceptually illustrated in Fig. 1a, and can be divided into two parts: the first part, connected to the input, is called the ‘reservoir’. The connectivity structure of the reservoir will remain fixed at all times (thus requiring no training); however, the neurons (network nodes) in the reservoir will evolve dynamically with the temporal input signals. The collective states of all neurons in the reservoir at time *t* form the reservoir state **x**(*t*). Through the dynamic evolutions of the neurons, the reservoir essentially maps the input **u**(*t*) to a new space represented by **x**(*t*) and performs a nonlinear transformation of the input. The different reservoir states obtained are then analyzed by the second part of the system, termed the ‘readout function’, which can be trained and is used to generate the final desired output **y**(*t*). Since training an RC system only involves training the connection weights (red arrows in the figure) in the readout function between the reservoir and the output^4^, training cost can be significantly reduced compared with conventional recurrent neural network (RNN) approaches^4^.

The readout function in an RC system is typically simple (thus easy to train) and is normally based on a linearly weighted combination of the reservoir neuron node values. As a result, it is memory-less. To process temporal information, the reservoir state needs to be determined not only by the present input but also by inputs within a certain period in the past. Therefore, the reservoir itself must have short-term memory. In fact, it has been mathematically shown^3^ that an RC system only needs to possess two very unrestrictive properties to achieve universal computation power for time-varying inputs: point-wise separation property for the reservoir, which means that all output-relevant differences in the input series **u**_**1**_(·) and **u**_**2**_(·) before time *t* are reflected in the corresponding reservoir internal states **x**_**1**_(·) and **x**_**2**_(·) that are separable; and approximation property for the readout function, which means that the readout function can map the current reservoir state to the desired current output with required accuracy.

Several studies have demonstrated the implementation of RC systems, using randomly connected atomic switches, field programmable gate arrays (FPGAs) and photonic systems^5–8^. Recent theoretical analyses on RC systems based on memristor devices have further shown that memristor-based RC systems can provide excellent performance in tasks, such as pattern recognition, signal processing and disease detection^9–12^ by taking advantage of the intrinsic nonlinearity and/or volatile (short-term memory) effects of the devices.

In this study, we experimentally demonstrate a memristor-based RC system using dynamic memristor devices that offer internal, short-term memory effects^13–15^. These dynamic effects allow the devices to map temporal input patterns into different reservoir states, represented by the collective memristor resistance states, which can then be further processed through a simple readout function. The memristor-based RC hardware system is then used to experimentally perform hand digit recognition tasks and solve a second-order nonlinear task.

## Results

### Short-term memristor dynamics

Memristors are two terminal resistive elements with memory effects, where the state of the device depends on one or more internal state variables and can be modulated depending on the history of external stimulation^16–19^. Generally speaking, a memristor’s resistance is determined by the internal ion (either anion or cation) configuration, where the re-distribution of oxygen ions or metal cations inside the device changes the local resistivity and the overall device resistance^17,19–21^. The compact device structure and the ability to both store and process information at the same physical locations make memristors and memristor crossbar arrays attractive candidates for neuromorphic computing applications^22–26^. At the single-device level, memristors have been shown to be able to emulate synaptic functions by storing analog synaptic weights and thus modulate the connection strength between the input and output neurons^22–24^, while recent studies have also demonstrated that these devices can even emulate synaptic effects faithfully based on internal ionic dynamics^13–15,27,28^.

Specifically, memristor devices with short-term memory effects^13–15^ are used in this study to act as the reservoir in an RC system. During device fabrication, the switching layer of the WO_*x*_ based device was specifically designed to exhibit short-term memory (i.e., volatile) behavior^13–15^ (see Methods section and Supplementary Fig. 1–5). To demonstrate the temporal dynamics of the device, write pulses having the same amplitude (1.4 V, 500 µs) are applied to the device at different timeframes and the response of the memristor, which is represented by the read current through a small read pulse (0.6 V, 500 µs) following each write pulse, is recorded. The results are shown in Fig. 1b. Two properties, similar to results obtained in dynamic synapses, can be observed: (1) if multiple pulses are applied with short intervals, the response will gradually increase (as indicated by the red arrow in the figure), showing an accumulation effect, (2) if there is a long enough period without any stimulation, then the device state will decay toward the original resting state, as indicated by the green arrow in the figure. This temporal response is attributed to the internal ionic processes of the WO_*x*_ memristor, including the drift under electric field during the spike and the spontaneous diffusion after the spike of oxygen vacancies, and can be well modeled within the memristor theoretical framework^13–15,28^. The memristor’s short-term memory effect can be described by a time constant *τ* (Supplementary Note 1 and Supplementary Fig. 5), which is around 50 ms for devices used in this study. As a result, when programming the device, the device state depends not only on the programming pulse itself, but also depends on whether other programming pulses have been applied in the immediate past within a period of around 50 ms. Prior programming pulses applied within this range will affect the device state, with pulses applied closer to present time having a stronger effect, while events happened much earlier will not affect the present device state since the device would have decayed to the initial state already.

### Realization of RC system for digit recognition

WO_*x*_ memristors selected from a 32 × 32 crossbar array (Supplementary Fig. 6) were used to form the reservoir, where each memristor device is connected to an input through a custom-built test board. After receiving temporal inputs, the memristor resistance values are measured using the test board and fed to the readout function. The uniformity and reliability of the devices in response to temporal inputs can be found in Supplementary Figs. 7,8. The readout function was implemented in software using Matlab (Methods section). The RC system is schematically shown in Fig. 1c.

We start with a simple task by processing computer generated images. The task is to recognize the digit from an input image, for example digit 2 from the image in Fig. 1d. The 5 × 4 image has 20 pixels, either black (0) or white (1). It is then divided into 5 rows, each row containing 4 consecutive pixels and is fed into a memristor in the reservoir as a 4-timeframe input stream. A timeframe (3 ms in width) will contain a write pulse (1.5 V, 1 ms) if the corresponding pixel is a white pixel, or no pulse (equivalently a pulse with amplitude of 0 V) if the corresponding pixel is a black pixel^29^. Therefore, information of the image for digit 2, which is represented by the spatial locations of the white pixels in each row, is represented by temporal features streamed into the reservoir, i.e., a pulse stream with pulses applied at different timeframes. The goal is to extract information of the image, i.e., the digit number 2 here, by collectively processing the temporal features in the 5 input pulse streams. Here only 5 memristors are used to process the image, with each memristor processing the input pulse stream from a specific row in the image. The reservoir state is represented by the collective resistance states of the 5 memristors. After the application of the input streams, the reservoir state is thus dependent on the input temporal patterns and can be used to analyze the input.

Specifically, when a pulse is applied, the state of the memristor will be changed (reflected as a conductance increase) and if multiple pulses are applied with short interval a larger increase in conductance will be achieved, while long intervals without stimulation will result in the memristor state (conductance) decaying toward its resting state, i.e., the initial state before any pulse is applied. Therefore, different temporal inputs will lead to different states of the device and consequently the overall reservoir state represented by all devices. In this specific set-up, each memristor’s state after stimulation will thus represent a specific feature for the given row in the original image, and the collective device states, representing the reservoir state, can be used to perform pattern recognition through the (trained) readout function, i.e., identifying the digit as 2 of the original input.

The readout function here is a 5 × 10 network, with the reservoir state, measured by the read currents from the 5 memristors in the reservoir, as the input, and 10 output neurons (labeled 0–9) representing the predicted digit value of the input image, schematically illustrated in Fig. 1c. During classification, the output from the 10 output neurons are calculated from the dot product of the 5 inputs and the weights associated with each output neuron, and the output with the maximum dot product is selected and its label number is used as the predicted digit value. The readout function is trained in a supervised fashion based on logistic regression (Methods section) where the weights are adjusted to minimize output error during training.

A significant advantage of using the RC system is the reduction of network size and training cost. A conventional neural network for this task will have 20 inputs corresponding to the 20 pixels and minimum 10 outputs. Even without any hidden layers, i.e., with the 20 inputs directly connected to the 10 outputs forming a 20 × 10 network, 200 weights need to be trained. This number will grow very quickly if one or more hidden layers are used. In the RC system, the spatial information is encoded in the temporal domain so a smaller network (e.g., a 5 × 10 readout function with only 50 weights) need to be trained, while the reservoir consisting of only 5 memristors does not need training.

### Training and classification of 5 × 4 images

**Fig. 2:**
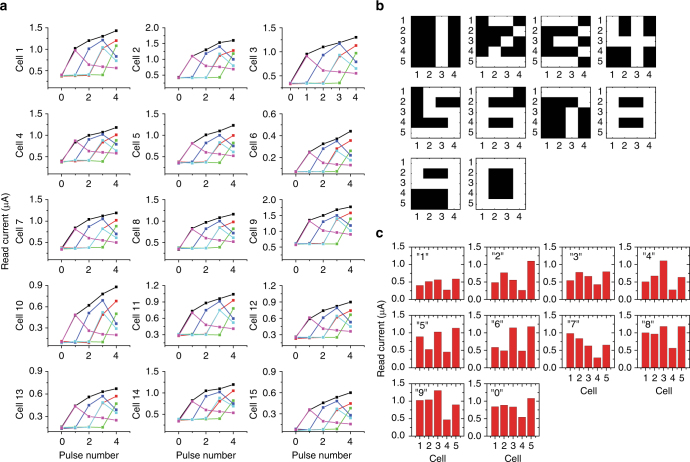
Reservoir states used to differentiate different temporal inputs. **a** The response of 15 memristors from the array to 6 different pulse streams (black: [1 1 1 1], purple: [1 0 0 0], blue: [0 1 1 0], red: [0 0 1 1], cyan: [0 0 1 0], green: [0 0 0 1]), showing similar response from all devices, as well as device variations. **b** Images of the 10 digits used in this test. **c** Experimentally measured reservoir states after the memristors are subjected to the 10 inputs. The reservoir state is reflected as the read currents of the 5 memristors forming the reservoir

Extensive tests were carried out to characterize the memristor response to different temporal inputs. Figure 2a shows results from one such test. Here 15 memristors are chosen from an array and their response to 6 different pulse streams are measured. Although there are some variations among the devices, all devices show the same trend when subjected to the different input pulse streams, and for all devices the read current (immediately after the pulse train) can be well separated for different inputs. For the 10 digits represented by the 5 × 4 images shown in Fig. 2b, there are overall 10 different pixel arrangements along each row direction, corresponding to 10 different possible pulse streams for the memristors. An example of a memristor responses to all 10 pulse stream configurations is shown in Supplementary Fig. 9, showing the memristor state can be used to separate these 10 inputs. The uniqueness of the memristor state for a given input was further verified by results shown in Supplementary Figs. 10,11. More details and discussions on memristor response to pulse streams can be found in Supplementary Note 2.

Figure 2c shows the reservoir state, represented by the combination of the 5 memristors’ resistance values, after feeding the reservoir with the 10 images shown in Fig. 2b. The reservoir states are significantly different, verifying the reservoir’s ability to clearly separate these 10 cases.

The reservoir state was then used as input to the readout network for training and classification. After 200 training iterations, the RC system can correctly recognize all inputs from the 10 original images. To test the effects of cycle-to-cycle variations of the device, the 10 images were repeatedly tested 10 times without retraining the readout function, and 100% accuracy was verified experimentally in the memristor-based RC system for this simple task.

**Fig. 3:**
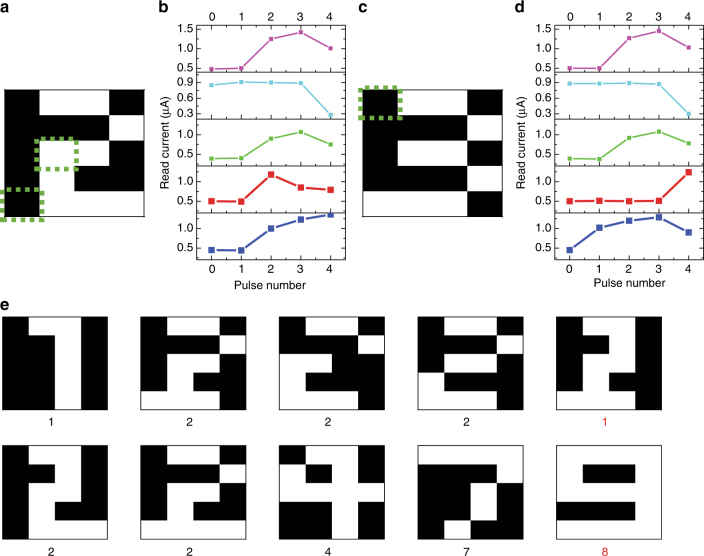
Recognition of noisy images. **a**, **c** Distorted images of digits 2 and 3 are generated by adding noise to the original data at locations marked by the dashed squares. **b**, **d** Corresponding reservoir states for these two inputs, showing differences in the two digits can be clearly captured by the memristors corresponding to the last two rows. **e** Recognition results of noisy digits. The RC system output, e.g., the predicted digit is shown below each case. The distorted images are generated by adding noise to the original training samples. The system can still successfully identify the majority of the distorted images without additional training, until too much noise is added as in the las two cases where the incorrect classifications are marked in red

The temporal information processing ability of the reservoir was more clearly revealed by testing images not included in the original training set. For example, two distorted images were generated by adding noise to digits 2 and 3 as shown in Fig. 3a, c (as marked by the dashed boxes). A close inspection will reveal that the number of pulses in the pulse streams (white pixels in each row) for these two digits are in fact identical for all rows (i.e., 2, 1, 2, 1, 3 pulses for rows 1–5). The only difference is the relative timing of the pulses in the last two rows. The reservoir states (shown in Fig. 3b, d) are sensitive to the temporal ordering of the pulses and the different temporal ordering in the last two rows in the two input cases leads to significantly different reservoir states (reflected in Fig. 3b, d, respectively), therefore enabling the reservoir to still separate these two different inputs and allow the system to successfully recognize the former as digit 2 while later as digit 3 through the readout network, without additional training.

We note that the noisy patterns were created by adding or removing one white pixel, but not simultaneously. This type of noise will thus have a smaller effect on the memristor response than re-ordering the pixels in the same row (as may be expected from different input classes such as the cases in Fig. 3a, c), and the output signal from the reservoir, although distorted, can still lead to successful classification by the readout function.

After adding more noise to the original images, the digits can still be recognized correctly by the system as shown in Fig. 3e. However, if too much noise is added, as in the last two examples shown in the figure, the system will no longer be able to recognize them without further training of the readout function. However, it could be argued that in these two cases, the noisy 2 can indeed by alternatively considered as a noisy 1, while the noisy 9 can in fact be considered as a noisy 8 (with a missing pixel) instead.

### MNIST data set classification

**Fig. 4:**
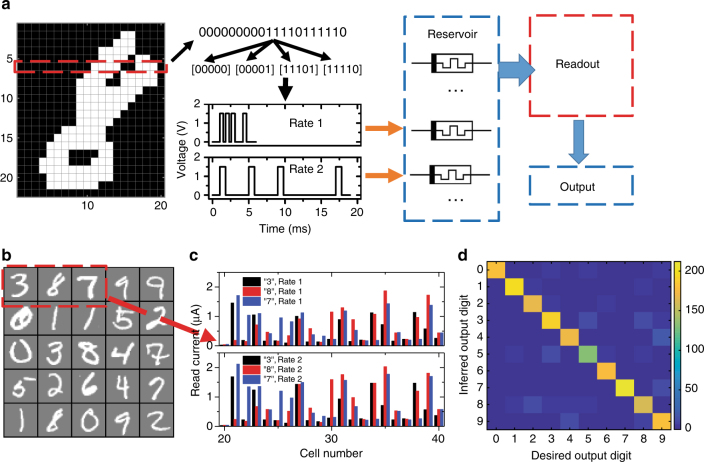
Handwritten digit recognition using a memristor-based RC system. **a** The process flow. The original digit image is first converted into pulse streams and fed to the memristor-based reservoir at different rates. The recognition result is generated after feeding the reservoir state to a trained readout function. **b** Some examples from the MNIST database. **c** Reservoir states corresponding to the three examples in **b** at two input rates (rate 1: timeframe width 0.8 ms, with pulse of 1.5 V, 0.5 ms; rate 2: timeframe width 4 ms, with pulses of 1.5 V, 0.8 ms), showing significant differences in the reservoir states. **d** False color confusion matrix showing the experimentally obtained classification results from the RC system vs. the desired outputs. The occurrence of the predicted output for each test case is represented by colors shown in the color scale. A recognition accuracy of 88.1% was obtained from the reservoir consisting of only 88 memristors

Following these demonstrations, the memristor-based RC system was then tested with a more complex, real-world task, that is, recognition of handwritten digits. We trained and tested the system with the commonly used Mixed National Institute of Standards and Technology database (MNIST, see Methods section)^30^. A preprocessing was performed before the images were fed into the reservoir, as shown in Fig. 4a. Take the image of digit 8 as an example, the original grayscale image was first converted into a binary-pixel image. The unused boarder area was also removed to reduce the original 28 × 28 image into a 22 × 20 image with 22 rows and 20 pixels per row. Some samples from MNIST are shown in Fig. 4b.

If the entire row is used as one input pulse stream then in theory there can be 2^20^ different input patterns which may be too difficult for one memristor to distinguish. Therefore, several optimization methods were introduced to improve the ability of the reservoir to separate the inputs. The first approach is to divide each row into smaller sections (e.g., 4 sections with each section now containing 5 pixels) to allow better separation of the inputs. Another strategy is to apply the same input as pulse streams at different rates (by using different timeframe widths). The rational is as follows. If the timeframe is short and thus the interval between pulses is small (compared to the decay time constant of the memristor), the increased conductance caused by each pulse will not decay much before the next pulse arrives. As a result, the final memristor conductance is largely determined by the number of pulses in the input due to the cumulative effects of the conductance increases. In the other extreme, if the timeframe is long the memristor will have enough time to decay toward the resting state, so the final memristor state is largely determined by pulses applied later in time. The relative timing between pulses will also have different effects in these cases, allowing the memristor-based reservoir to perform different transformations of the temporal information in the input to allow better separation of the reservoir states. Equivalently, similar effects can be obtained by applying copies of input pulse streams to memristors with different internal time constants. In this study, we used pulses with different timeframe widths applied to (nominally) identical memristors out of convenience.

With these considerations, the image is fed into the reservoir in 5 pixel sections as input pulse streams and applied with two different rates, as shown in Fig. 4a. The readout network is trained using logistic regression as discussed earlier. Fourteen thousand images from the MNIST data set was used for the readout function training. After training, another set of samples consisting of 2000 images not in the training set, are used to test the recognition accuracy. Figure 4c shows the experimentally measured reservoir states corresponding to the three test samples shown in Fig. 4b at two different input rates, demonstrating that significant difference can be achieved in the reservoir to allow effective separation of the inputs and subsequent classification in the readout network. The reservoir state was then fed to the readout function to perform classification. In the experimental study, 88 memristors were used as the reservoir (22 rows, 4 sections and 2 rates), and a 176 × 10 readout network was used for classification. From the 2000 test images, an 88.1% accuracy was obtained from the RC system. Figure 4d shows a false color confusion matrix highlighting the experimentally obtained classification results from the RC system vs. the desired outputs. If the inputs were fed to the reservoir at only one rate, an 85.6% recognition accuracy was obtained experimentally, supporting the hypothesis that input with more than one rate improves the reservoir’s ability to process temporal information.

The memristor-based RC system was further analyzed through simulation using a physics-based memristor model (details are shown in Supplementary Note 3 and Supplementary Tables 1–4). From simulations based on the dynamic WO_*x*_ memristor model^15^, an RC system with a reservoir consisting of 88 memristor devices (22 rows, each row has 4 sections and each section is input at 2 rates) can potentially achieve 91.1% recognition accuracy. Increasing the reservoir to 112 memristors (28 rows, 4 sections, 3 rates) improves the performance slightly to 91.5% accuracy. The lower accuracy obtained in the experimental network can be attributed to the cycle-to-cycle variations of the device response, during training and the image analysis stages. We note that even with these non-idealities, the experimental results, with a much smaller network and dealing with a simplified, truncated input, are already better than the 88% accuracy achieved previously by simulation based on a one-layer neural network with 7850 free parameters, using pixel values of the entire digit image as the input^31^. Additional benchmarking analysis against a conventional approach with an added hidden layer to achieve the same connectivity pattern as the RC system show that for a given readout network size, the RC system generally outperforms the conventional network system, particularly at smaller network sizes (Supplementary Note 4 and Supplementary Figs. 12, 13).

### Solving a second-order nonlinear dynamic task

In the experiments of digit image recognition, we partitioned the two-dimensional images row-wise and converted spatial patterns into temporal inputs to the reservoir. More native applications of the reservoir system may be to perform temporal data directly, i.e., analyzing time series data and solving dynamic nonlinear problems. Below we show another experiment where the memristor-based reservoir hardware system is used to solve a second-order dynamic nonlinear task.

**Fig. 5:**
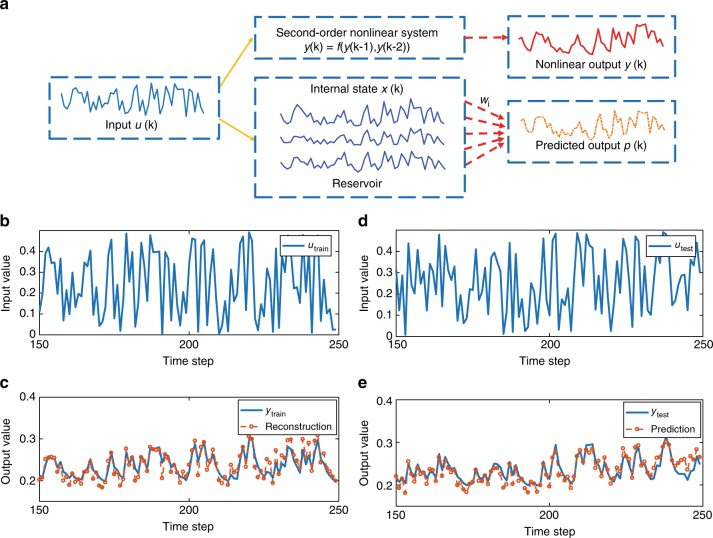
Solving a second-order nonlinear dynamic task. **a** Schematic showing how the memristor reservoir can be used to map an unknown nonlinear dynamic system. The original input signals *u*(*k*) are fed into the original second-order nonlinear system and the output signals *y*(*k*) are generated (upper branch). The same inputs when fed into a memristor reservoir can generate different reservoir states, which are in turn used by the readout function to produce the predicted output *p*(*k*). **b** Uniform random signals *u*_train_(*k*) are used as the training input. **c** Theoretical output *y*(*k*) (blue solid line) vs. experimentally reconstructed output *p*(*k*) from the memristor reservoir computing system (red circles and dashed line), for 100 timeframes from the random training set. **d** Another set of uniform random signals *u*_test_(*k*) are used to test the RC performance. **e** Theoretical output *y*(*k*) (blue solid line) vs. experimentally reconstructed output *p*(*k*) from the memristor RC system (red circles and dashed line), for 100 timeframes from the random untrained test set. The readout function was not re-trained in the test

Nonlinear dynamical systems are commonly used in electrical, mechanical, control, and other engineering fields^32^. Among which, second-order nonlinear dynamic systems are widely studied as a model system because of their close relations to electrical systems (i.e., RLC circuits). Figure 5a shows the schematic of using an RC system to map a second-order dynamic nonlinear system. For a given input *u*(*k*) at timeframe *k*, the system generates an output *y*(*k*) following a nonlinear transfer function that may have a time lag. In our experiment, we choose a second-order dynamic nonlinear transfer function following a prior study^33^, described as:

1 $$y\left( k \right) = 0.4y\left( {k - 1} \right) + 0.4y\left( {k - 1} \right)y\left( {k - 2} \right) + 0.6u^3\left( k \right) + 0.1.$$

As can be observed from equation (1), the output *y*(*k*) at timeframe *k* not only depends on the current input *u*(*k*), but is also related to the cross term of past two outputs, y(*k*–1) and *y*(*k*–2) at timeframes *k*–1 and *k*–2, which makes it a second-order nonlinear system with a time-lag of two time-steps. In typical applications, the relationship between *y*(*k*) and *u*(*k*) is implicit and hidden, which makes the problem difficult to solve. For example, an attempt to solve this problem with a conventional network shows large error for both the training and testing data (Supplementary Note 5, Supplementary Fig. 14).

The goal is to train the memristor-based RC system to map the hidden nonlinear transfer function, so the correct output *y*(*k*) can be obtained from the input *u*(*k*) after training, without knowing the original expression between *u*(*k*) and *y*(*k*).

We note this type of nonlinear problems are well suited for reservoir systems such as the one presented here, since each output *y*(*k*) is dependent on the recent past results but not on the far past, matching well with systems having short-term memory effects. We use a 300 timeframe-long random sequence as inputs to train the memristor-based RC system (Methods sections). The training signal is shown in Fig. 5b. The reservoir consists of 90 physical memristor devices chosen from the memristor crossbar array, and is divided into 10 groups with 9 devices in each group. Input voltage pulse streams with 10 different timeframe widths (1 ms, 2 ms, 3 ms, 4 ms, 5 ms, 6 ms, 8 ms, 10 ms, 15 ms, and 20 ms) are then, respectively, applied to the 10 groups through the test board. We found having 9 devices in each group improves the reservoir performance (Supplementary Fig. 15) due to inherent device variations that help make the reservoir output more separable, as well as having inputs with different timeframe widths as has already been discussed in the MNIST case. The readout layer in this case is a 90 × 1 feedforward layer, and is used to convert the reservoir state to a single output *y*(*k*). A simple linear regression training algorithm based on batch gradient descent is used to train the readout function (Methods section).

Figure 5c shows the experimentally obtained reconstructed (i.e., predicted) outputs from the physical memristor RC system after training (red cycles and dashed line), and the theoretical output (i.e., ground truth) *y*(*k*) (blue solid line) from the training sequence, showing the memristor RC system can correctly solve the dynamic nonlinear problem, with a normalized mean squared error (NMSE, Methods section) of 3.61 × 10^−3^. More importantly, to verify the memristor RC system has indeed solved the dynamic transfer function, we tested the system using a new, independently generated random sequence (Fig. 5d) other than the training sequence. Figure 5e shows that the system is still able to successfully predict the expected dynamic output for the random, untrained sequence using the same readout function, with a similar NMSE of 3.13 × 10^−3^.

## Discussion

In this study, we demonstrate a memristor-based RC system by utilizing the internal, short-term ionic dynamics of memristor devices. We show experimentally that even a small reservoir consisting of 88 memristor devices can be used to process real-world problems such as handwritten digit recognition with performance comparable to those achieved in much larger networks. A similar-sized network is also used to solve a second-order nonlinear dynamic problem and is able to successfully predict the expected dynamic output without knowing the form of the transfer function.

It should be noted that the system is not fully optimized for the handwritten digit recognition task yet so the performance could still be improved further. First, information from the original data has already been partially lost during the preprocessing, such as transforming the grayscale image to binary data. Second, the pulse amplitude, width and rates could still be fine-tuned to maximum classification yield. Additionally, while normal neural networks aim to extract features across the image from several rows simultaneously, the reservoir presented here only processes each row separately and independently. A quick solution would be to scan the digit also in the vertical direction and input each column to the reservoir to allow relations between the rows to be processed by the reservoir as well. Indeed, adding vertical scan can improve the classification accuracy to 92.1% as verified through simulation using the device model (Supplementary Table 3), although the system also becomes larger and requires 672 inputs.

The computing capacity added by the memristor-based reservoir layer was analyzed by comparing the RC system performance with networks having the same connectivity patterns, by replacing the reservoir layer with a conventional nonlinear downsampling function (Supplementary Note 4, Supplementary Figs. 12–13). The RC system outperforms the conventional approach and the advantage is significant at small readout network sizes, even for the image analysis task that is not naturally fitted for RC. For the second-order dynamic problem that is more naturally suited for the RC system, our analysis shows that the small RC system significantly outperforms a conventional linear network, with orders-of-magnitude improvements in prediction NMSE (Supplementary Fig. 15). We also show that the inherent device variations, which can pose significant challenges for some applications, become a benefit for RC systems, as they help make the reservoir states more separable (Supplementary Fig. 15)^11,29^.

The demonstration of memristor-based RC systems will stimulate continued developments to further optimize the network performance toward broad applications in areas, such as speech analysis, action recognition and prediction. This approach will also be attractive for applications that do not require fast processing speed but have strong constraints on memory size and computation power. Finally, we want to note that the crossbar used in this work mainly provides the high-density devices, and the devices function independently in the reservoir since the short-term memory property is a native property of the device itself. Future algorithm and experimental advances that can take full advantage of the interconnected nature of the crossbar structures, by utilizing the intrinsic sneak paths and possible loops in the system may further enhance the computing capacity of the system.

## Methods

### Memristor array fabrication

The array of WO_*x*_ devices were fabricated following our previous approaches. Briefly, 60 nm of W was first sputter deposited on a Si carrier wafer with a 100 nm thermally grown oxide. The bottom electrodes (BEs) with 500 nm width were patterned by e-beam lithography and reactive ion etching (RIE) using Ni as a hard mask. Afterwards, the Ni hard mask was removed by wet etching. 250 nm of SiO_2_ was then deposited by plasma-enhanced chemical vapor deposition, followed by RIE etch back to form a spacer structure along the sidewalls of the BEs. The spacer structure allows better step coverage of the top electrodes (TEs) at the crosspoints and also restricts the resistive switching regions to a flat surface. The resistive switching WO_*x*_ layer was formed via rapid thermal annealing of the exposed W electrode surface with oxygen gas at 375 °C for 60 s. Afterwards, the TEs (Pd (90 nm)/Au (50 nm)) were patterned by e-beam lithography, e-beam evaporation and liftoff processes. Another RIE process was used to remove the WO_*x*_ between the TEs to isolate the devices and to expose the BEs for electrical contacts. Finally, a photolithography, e-beam evaporation and liftoff process was performed to form wire bonding pads of 150 nm thick Au. Supplementary Fig. 1 shows a schematic of the memristor structure. With the W bottom electrode partially oxidized to form the nonstoichiometric WO_*x*_ switching layer, and Pd/Au top electrode. Supplementary Fig. 2 shows a scanning-electron microscopy (SEM) image of a fabricated 32 × 32 memristor array. After fabrication, the memristor chip was then wire bonded to a chip carrier and mounted on a customized board for electrical testing.

### Mixed National Institute of Standards and Technology database

The data set, Mixed National Institute of Standards and Technology (MNIST) database^30^ is a large database that is commonly used for training and testing in the field of machine learning. The database was created by “remixing” the digit samples written by high school students and employees of the United States Census Bureau, and consists of 60,000 training samples and 10,000 test samples.

### Experimental set-up for RC system

The memristor measurement is performed on a custom-designed PCB board. It can measure crossbar arrays up to 32 rows and 32 columns. The board contains four Digital-to-Analog Converters (DACs) capable of producing 0–5 V independently and two Analog-to-Digital Converters (ADCs) to measurement current. The board is capable of performing tests including DC sweeps and pulse measurements.

Eighty-eight memristors were selected from the 32 × 32 crossbar array for the handwritten digit recognition test. The devices were selected in a way to avoid having adjacent devices in both row and column direction to minimize the write disturbance (Supplementary Fig. 6). Each 22 × 20 training image was converted into 88 pulse streams, with each row represented by 4 pulse streams. The pulses streams were then applied to the 88 devices. The device states after the pulse trains were measured and recorded. After each training image, the devices were erased to their initial states, and the process was repeated. The reservoir states recorded from the 14,000 training images, were used to train the readout function.

During the reservoir operation, we apply one pulse stream to one device at a time, by apply the voltage pulses to the row of the selected device in the memristor array and keeping the column grounded. Other rows and columns are also grounded.

### Readout function training via logistic regression

A supervised learning algorithm, logistic regression, was used to train the readout functions for the simple digit recognition task shown in Figs. 2, 3 and for the handwritten digit recognition task in Fig. 4.

Suppose the reservoir state is **x**, which is represented by a vector containing 5 elements (the 5 memristor conductance values) for the network used in Figs. 2, 3. The vector representing the reservoir state is applied to the readout network. The probability of the reservoir state corresponding to the different possible outputs is determined by the input vector and the weight matrix **θ** ^34^)

2 $$h_\theta \left( {\mathbf{x}} \right) = g\left( {{\mathbf{\theta }}^{\mathrm{T}} \cdot {\mathbf{x}}} \right),$$

3 $$g\left( {\mathbf{z}} \right) = \frac{1}{{1 + \mathrm{e}^{ - {\mathbf{z}}}}}.$$

The cost function defined is as

4 $$J\left( {\mathbf{\theta }} \right) = \frac{1}{m}\mathop {\sum }\limits_{i = 1}^m \left[ { - {\mathbf{y}}^{\left( i \right)}{\rm log}\left( {h_\theta \left( {{\mathbf{x}}^{\left( i \right)}} \right)} \right) - \left( {1 - {\mathbf{y}}^{\left( i \right)}} \right){\rm log}\left( {1 - h_\theta \left( {{\mathbf{x}}^{\left( i \right)}} \right)} \right)} \right],$$

where *m* is the number of samples, **y** ^(*i*)^ is the desired output for input **x** ^(*i*)^.

To minimize the cost function, the network is trained using the gradient descent defined as

5 $$\frac{{\partial J\left( {\mathbf{\theta }} \right)}}{{\partial {\mathbf{\theta }}_j}} = \frac{1}{m}\mathop {\sum }\limits_{i = 1}^m \left( {h_\theta \left( {{\mathbf{x}}^{\left( i \right)}} \right) - {\mathbf{y}}^{\left( i \right)}} \right){\mathbf{x}}_j^{\left( i \right)}.$$

The training of weights is achieved in Matlab 2016b using function *fmincg()*, which was provided by Jason Rebello as a logistic regression routine with regularization and has been commonly used to classify handwritten digits.

The same approach was used to train the readout function for the handwritten digit recognition task in Fig. 4.

### Training and testing the second-order nonlinear task

Random sequences based on uniform random distribution were used to train and test the memristor RC system for the second-order dynamic task implementation:

6 $$u\left( k \right) = {\rm rand}\left[ {0,0.5} \right].$$

The amplitude of the input signal *u(k)* is linearly converted into a voltage pulse with amplitude *V(k)* that is then applied to the memristor reservoir:

7 $$V\left( k \right) = 2{\mathrm{*}}u\left( k \right) + 0.8.$$

This linear conversion allows the input voltage pulses to fall in the range of 0.8–1.8 V for memristor stimulation. After collecting the reservoir output, the data are fed into the readout function. Following a similar approach in a prior study^35^, we ignore the first 50 initial data points in the transient period and train the readout function weights *w*_*i*_ (*i *= 1,…90) using the last 250 points in the training sequence using simple linear regression. The same training procedure is also applied for the linear network case used for comparison analysis.

### Readout function training via linear regression

A supervised learning algorithm, linear regression, was used to train the readout functions for the dynamic task shown in Fig. 5.

Suppose the reservoir state is ***x***, which is represented by a vector containing *n* elements (the conductance values of the *n* memristors forming the reservoir). The vector representing the reservoir state is applied to the readout network.

The cost function is defined as

8 $$J\left( {\mathbf{\theta }} \right) = \frac{1}{{2m}}\mathop {\sum }\limits_{i = 1}^m \left( {{\mathbf{\theta }}^{\mathrm{T}}{\mathbf{x}}^{\left( i \right)} - {\mathbf{y}}^{(i)}} \right)^2,$$

where *m* is the number of samples, **y** ^(*i*)^ is the desired output for input **x** ^(*i*)^.

To minimize the cost function, the network is trained using the gradient descent defined as

9 $$\frac{{\partial J\left( {\mathbf{\theta }} \right)}}{{\partial {\mathbf{\theta }}_j}} = \frac{1}{m}\mathop {\sum }\limits_{i = 1}^m \left( {{\mathbf{\theta }}^{\mathrm{T}}{\mathbf{x}}^{\left( i \right)} - {\mathbf{y}}^{(i)}} \right){\mathbf{x}}_j^{\left( i \right)}.$$

The training of weights is achieved in Matlab 2016b. Training typically takes 2000 iterations.

### Normalized mean squared error

We calculate our output signal error using the normalized mean squared error (NMSE), which is defined as following:

10 $${\rm NMSE} = \frac{{\mathop {\sum }\nolimits_k \mathop {\sum }\nolimits_i \left( {p_i\left( k \right) - y_i\left( k \right)} \right)^2}}{{\mathop {\sum }\nolimits_k \mathop {\sum }\nolimits_i y_i^2(k)}},$$

where *p*(*k*) is the predicted signal and *y*(*k*) is the original signal. Since the result is normalized by the original signal, the error is unitless.

### Data availability

All relevant data are available from the authors.

## Electronic supplementary material

Supplementary Information
