# Supplementary material for: Reservoir computing using dynamic memristors for temporal information processing
Source: Nat Commun. 2017 Dec 19;8:2204. doi: 10.1038/s41467-017-02337-y (PMC5736649; doi:10.1038/s41467-017-02337-y)
Supplement: Supplementary file 1 — Supplementary Information [file 41467_2017_2337_MOESM1_ESM.pdf]

Supplementary Figures

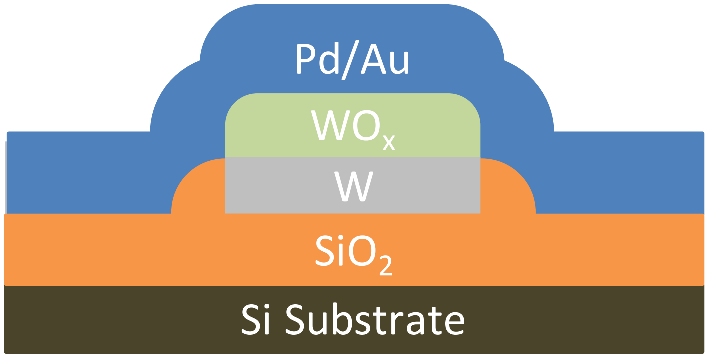

Supplementary Figure 1 | Schematic of the WO_x memristor with a metal-insulator-metal structure.

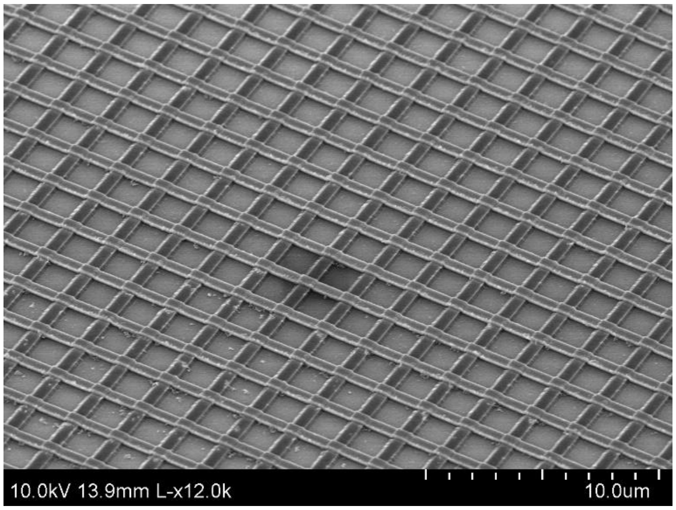

Supplementary Figure 2 | SEM image of a fabricated 32×32 WO_x memrsitor array. The reservoirs of the RC systems are built using several cells selected from the array.

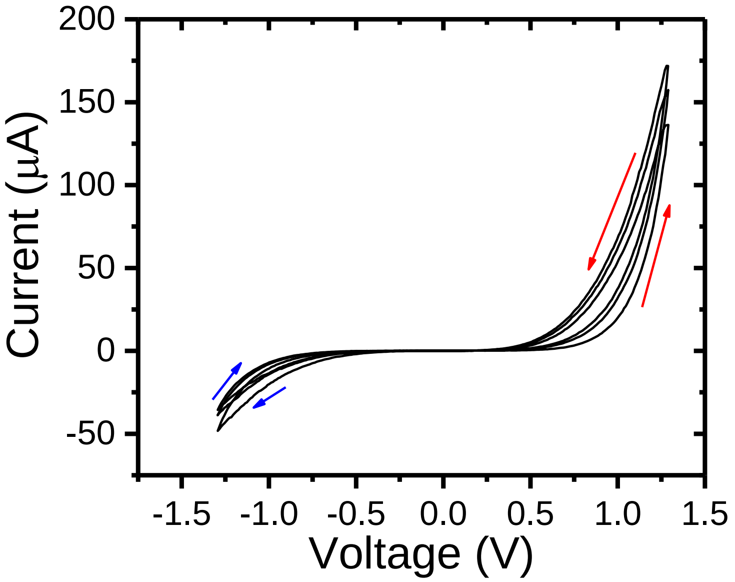

Supplementary Figure 3 | DC voltage sweeps on a WO_x memristor, showing gradual state changes. The device conductance was increased during the 3 consecutive positive sweeps (red arrows), then decreased during the 3 subsequent negative sweeps (blue arrows).

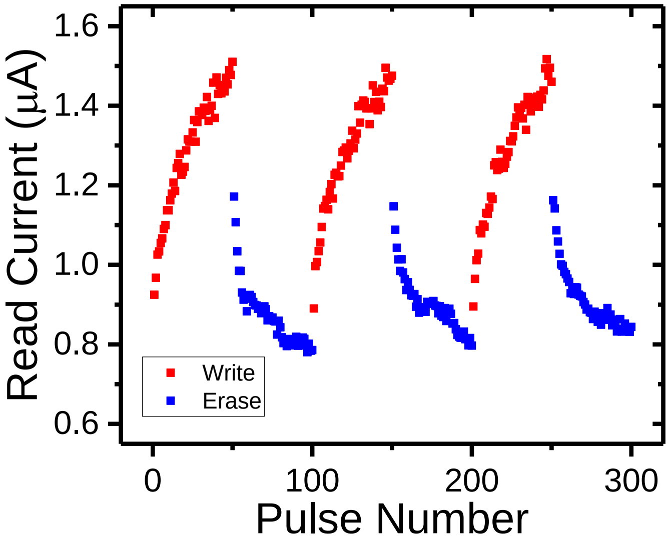

Supplementary Figure 4 | Pulse measurements of a WO_x memristor, showing the gradual conductance changes. Positive write pulses (+1.4 V, 100 μs) gradually increase the device conductance (red squares) while negative erase pulses (-1.3V, 100 μs) gradually decrease the conductance (blue squares).

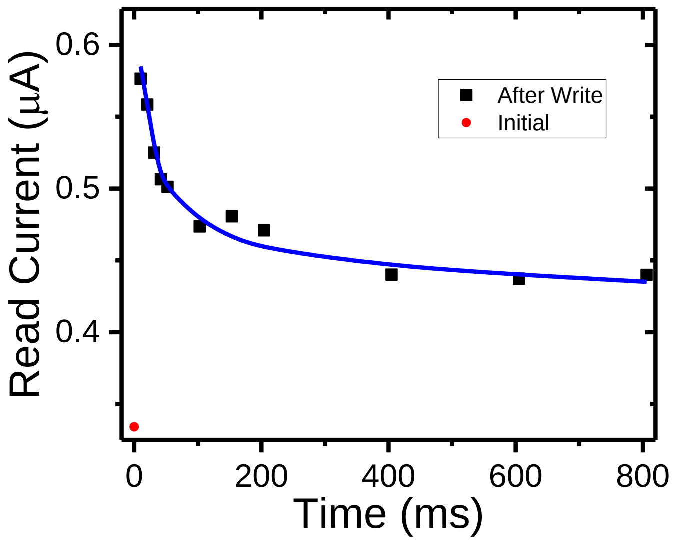

Supplementary Figure 5 | Conductance decay in WO_x memristor. The device was first programmed by 5 write pulses (1.4 V, 1 ms) then its conductance was monitored by periodic read pulses (0.4 V, 500 μs).

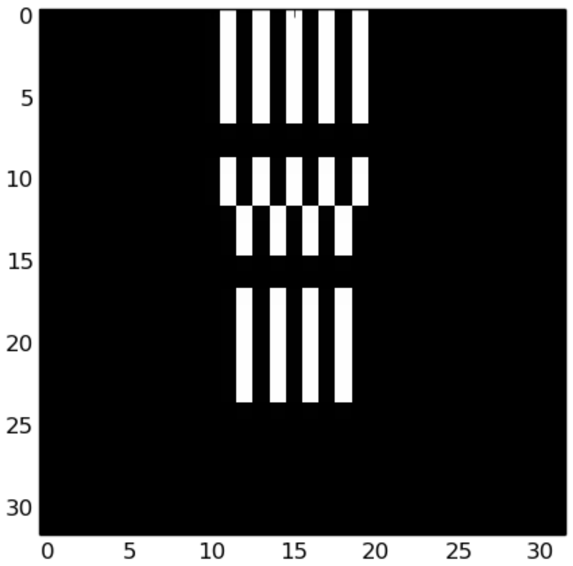

Supplementary Figure 6 | Locations of all 90 devices used in Figure 5. The devices are chosen in a way to avoid having adjacent devices in both row and column direction to minimize the write disturbance

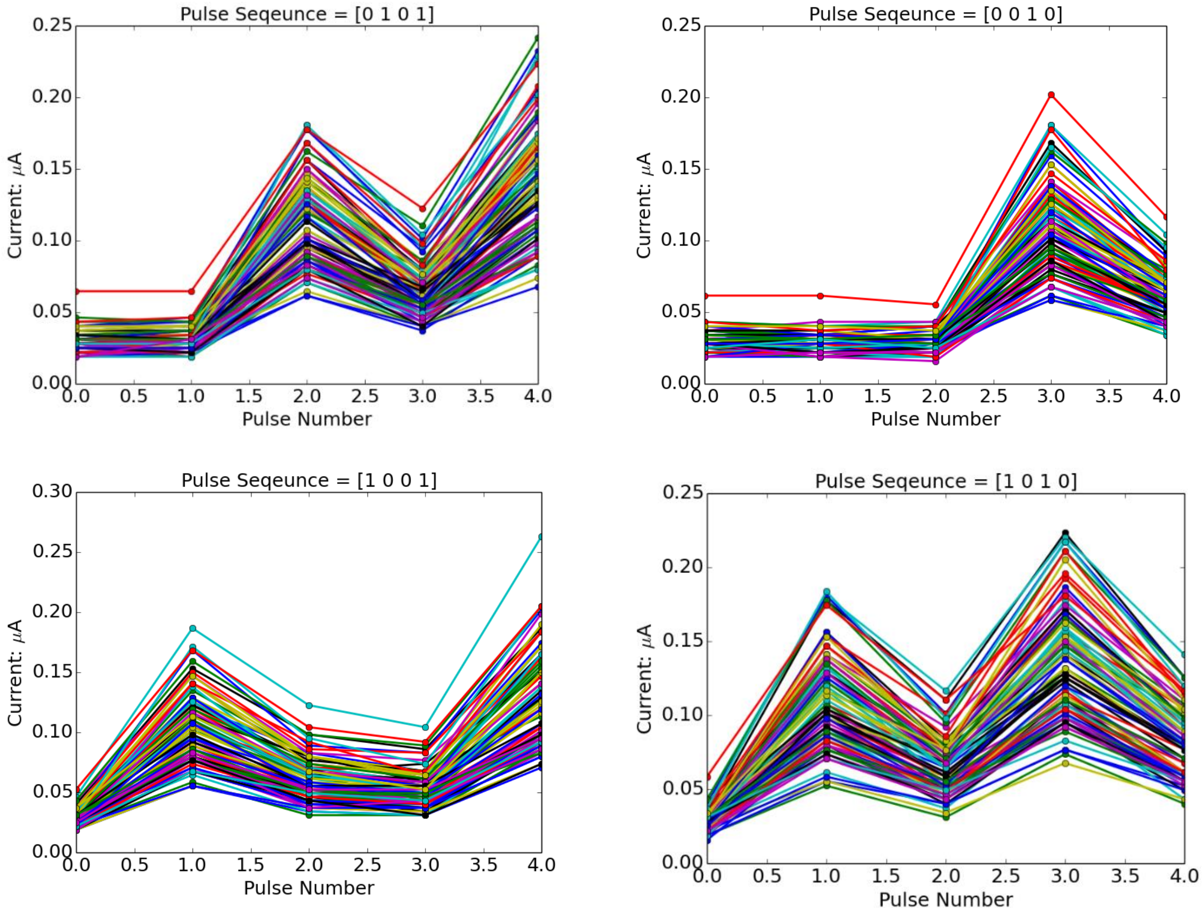

Supplementary Figure 7 | Response from the 90 devices to four different input pulse sequences. All devices demonstrate similar response to the input pulse streams.

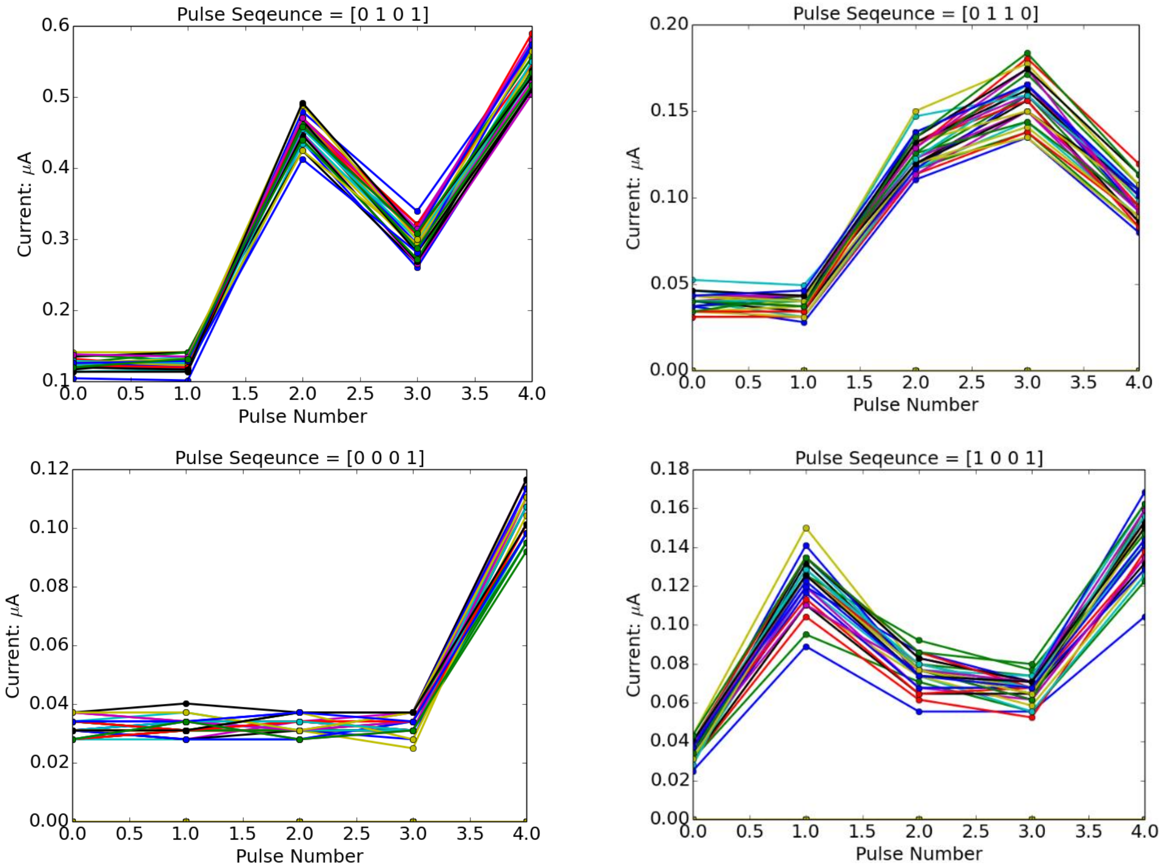

Supplementary Figure 8 | Response from a single device to the same input pulse streams, repeated 30 times in each test. The device shows similar response to each input pulse stream.

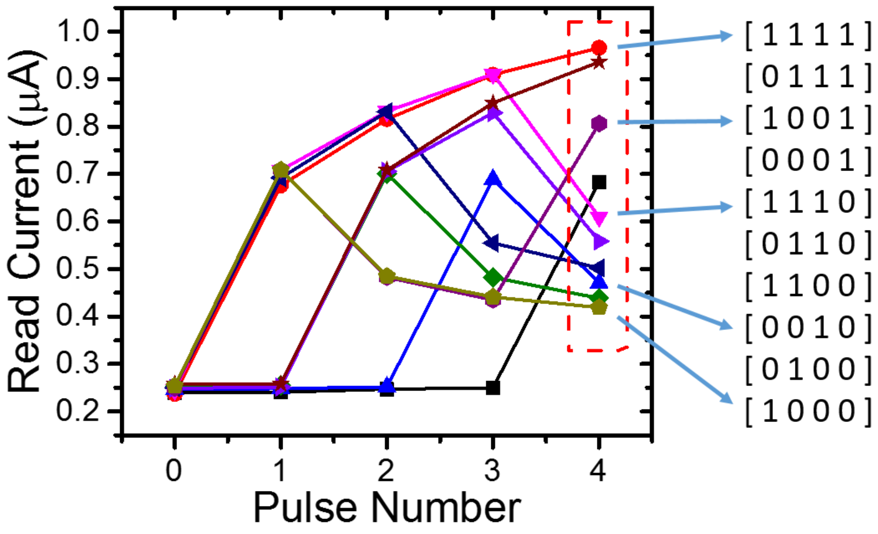

Supplementary Figure 9 | Memristor's response to ten different pulse streams. These pulse streams correspond to all possible different row pixel arrangements for the images shown in Figure 2b. The memristor state, reflected as the read current after each pulse stream, shows ten different levels that can be well separated.

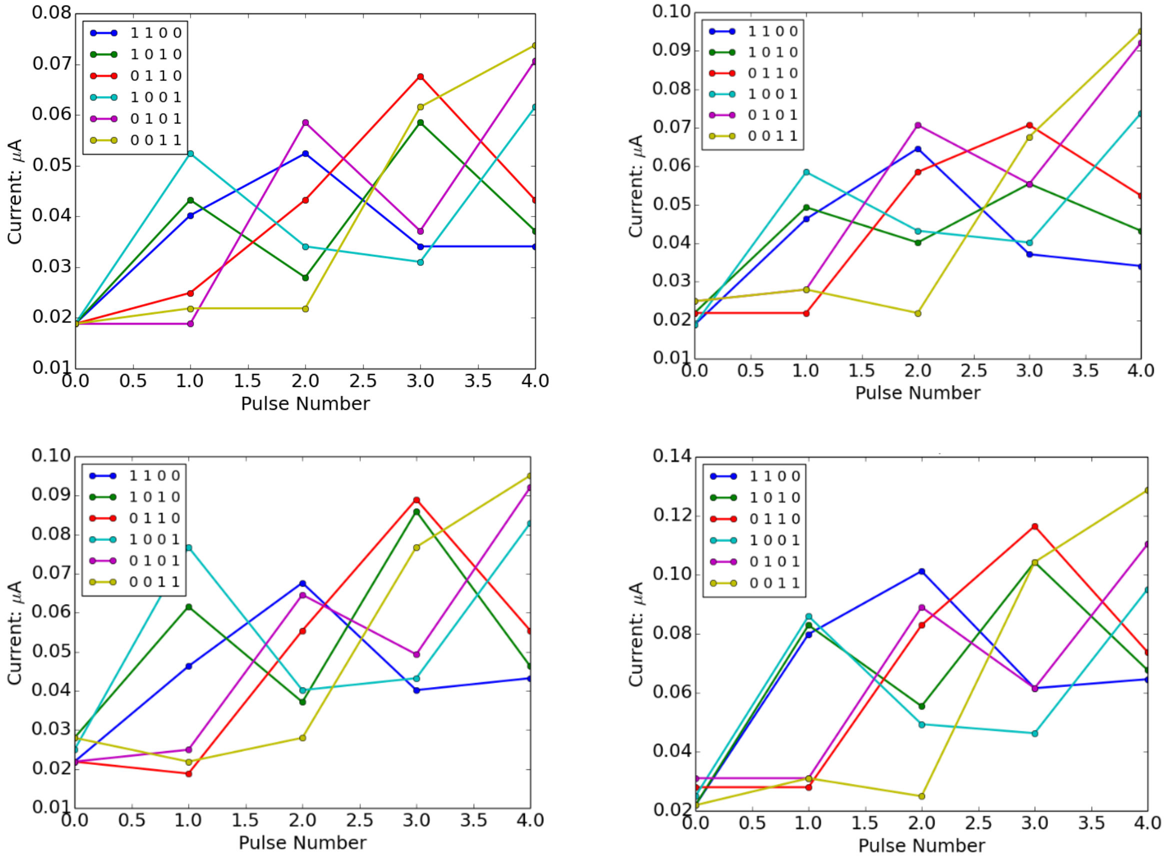

Supplementary Figure 10 | Response of 4 memristor devices to different variations of inputs having 2 ONEs and 2 ZEROs, showing the ability of the device to recognize temporal ordering of the inputs due to the short-term memory effects of the device.

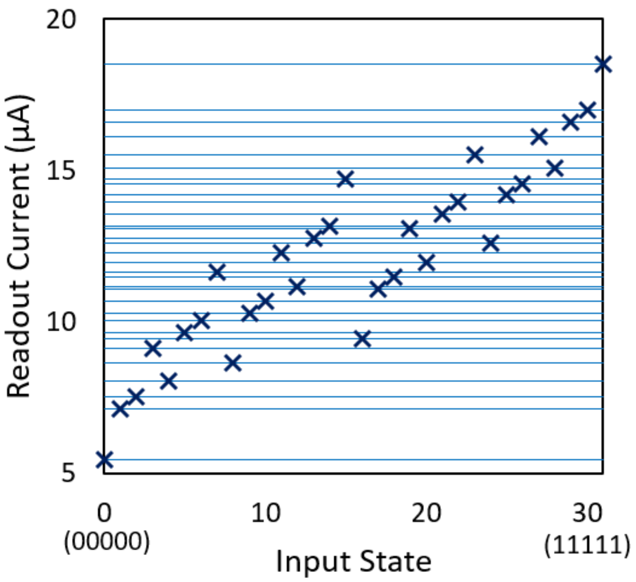

Supplementary Figure 11 | The uniqueness of the output for a given input is further verified by simulations using a realistic device model. The simulation uses a timeframe width of 10ms and spans all possible input scenarios.

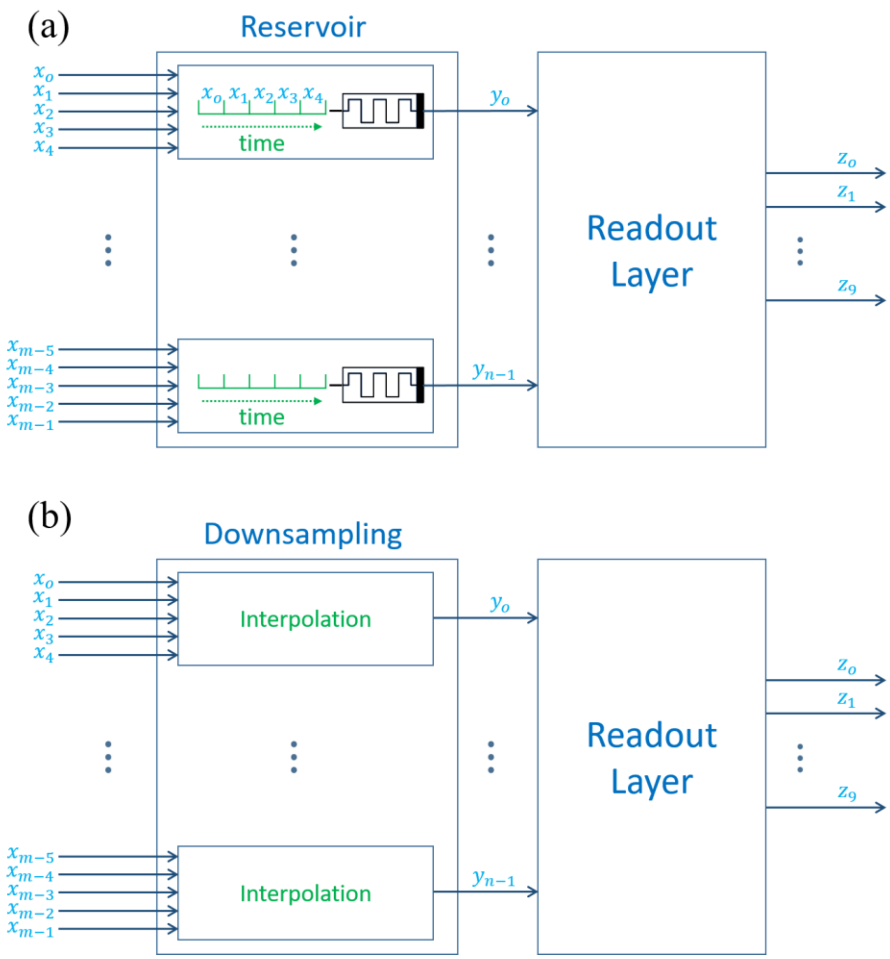

Supplementary Figure 12 | Comparison of the RC system having a memristor reservoir and a readout function (a), and a conventional network having a downsampling function and a similarly sized readout function layer (b). The downsampling function in (b) uses cubic interpolation to compress a group of multiple inputs into a single output that is then fed to the readout function.

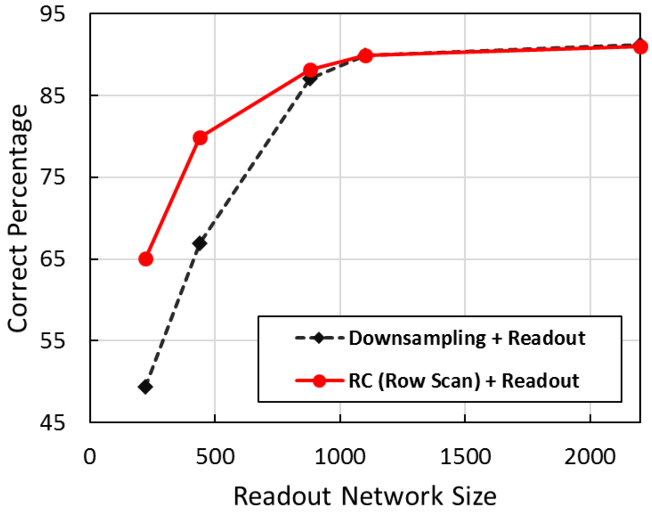

Supplementary Figure 13 | MNIST task performance vs. network size for the RC system and for the conventional network with a downsampling function. The original images (22x20) were reduced into sizes of 22x1, 22x2, 22x4, 22x5, and 22x10 through either the memristor reservoir layer or the downsampling layer and then fed to the readout function. The readout layer has 10 outputs corresponding to the 10 digit labels. The results were obtained through simulation using Matlab, based on the standard cubic interpolation downsampling function and a realistic memristor model as discussed in Supplementary Reference [2]

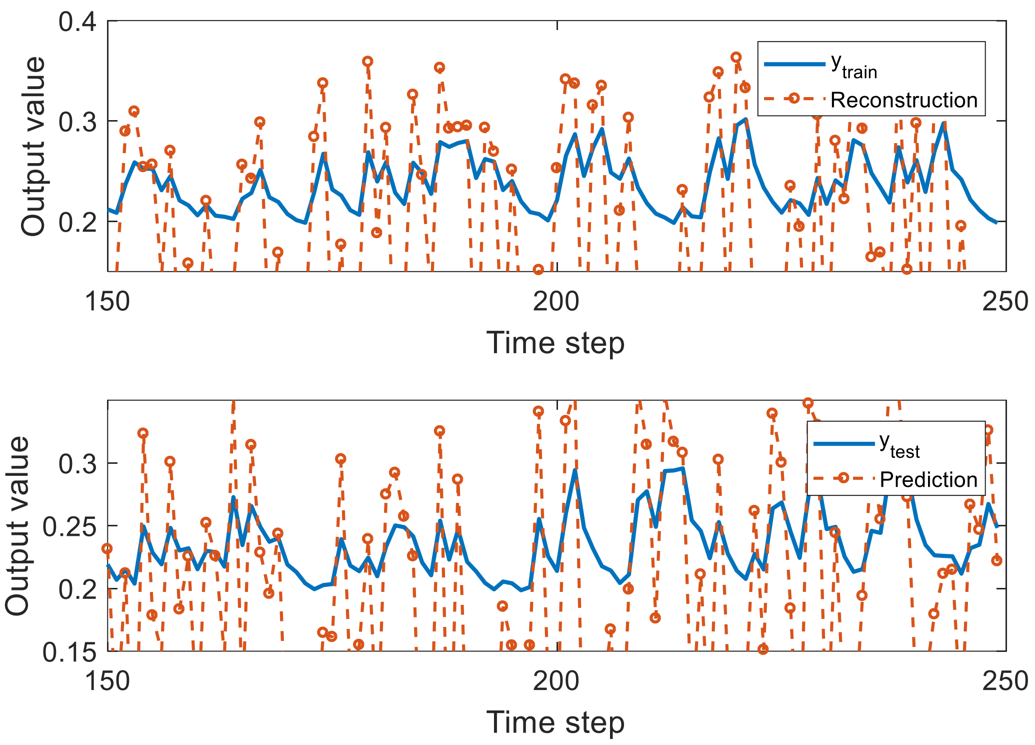

Supplementary Figure 14 | 100 points of the theoretical (blue solid line) and experimental reconstructed outputs (red circle line) of the training data (upper plot) and test data (lower plot) are shown for the conventional network.

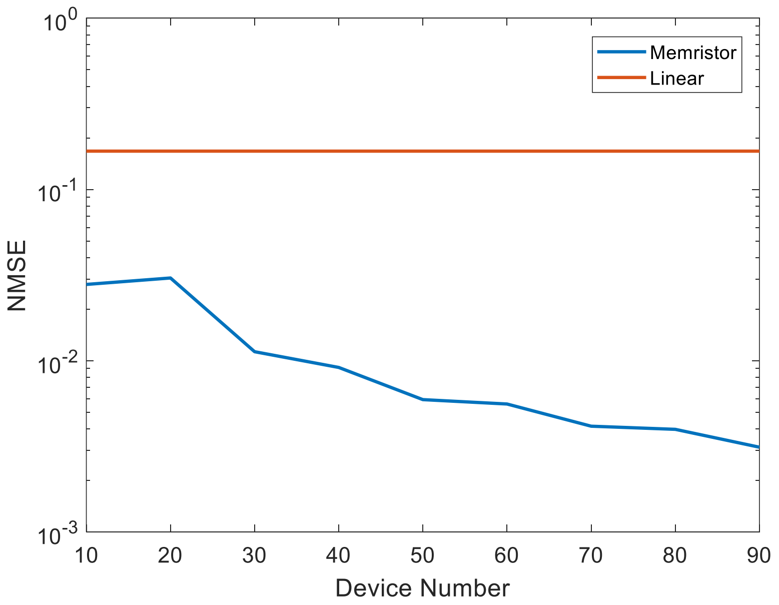

Supplementary Figure 15 | Comparison of the NMSE between the memristor RC system and the conventional linear network. The number of devices in each group in the reservoir layer is increased from 1 to 9 in these tests. The reservoir consists of 10 such groups.

Supplementary Tables

Parameters	Value	Parameters	Value
α	1e-8	λ	1e-3
β	0.5	η	8
γ	1e-5	τ	0.05
δ	4.0		

Supplementary Table 1 | Parameters of the WO_x memristor model used in the simulation.

<table>
<tr><td>Write pulse</td><td colspan="2">1.5 V, 1 ms</td></tr>
<tr><td>Read pulse</td><td colspan="2">0.6 V, 500 μs</td></tr>
<tr><td rowspan="3">Input rates</td><td>3 rates</td><td>500 Hz, 100 Hz, 20 Hz</td></tr>
<tr><td>2 rates</td><td>500 Hz, 20 Hz (yielding best recognition results)</td></tr>
<tr><td>1 rate</td><td>100 Hz (yielding best recognition results)</td></tr>
</table>

Supplementary Table 2 | Parameters used during the MNIST recognition simulation.

	Data Preprocessing	Number of samples	Sections of Each Row	Rates	Recognition Accuracy (%)
Simulation	Initial images 28×28	Training: 60000<br>Test: 10000	4	3 horizontal + 3 vertical	92.1
				3	91.5
				2	91.0
				1	86.0
	Truncating initial image size to 22×20	Training: 60000<br>Test: 10000	4	2	91.1
				1	88.2
			2	2	89.0
				1	79.9
Experiment	Truncating initial image size to 22×20	Training: 14000<br>Test: 2000	4	2	88.1
				1	85.6

Supplementary Table 3 | Experimental and simulation results of the MNIST handwritten digit recognition task using the memristor-based RC system. Better recognition can be achieved with more sections for each row and using more input rates for each section.

Inferred Output

**0**	181 (190)	0	0	4	0	5	0	0	0	4
**1**	0	197 (210)	4	3	1	1	2	3	6	1
**2**	1	2	167 (190)	6	0	0	4	1	3	1
**3**	2	1	2	190 (225)	0	11	0	1	11	4
**4**	1	0	2	0	172 (192)	2	1	3	3	18
**5**	3	4	1	12	1	130 (156)	5	1	6	1
**6**	0	1	1	0	2	3	177 (193)	0	0	0
**7**	2	0	2	2	0	0	1	212 (222)	2	11
**8**	0	5	10	6	4	4	3	0	160 (200)	3
**9**	0	0	1	2	12	0	0	1	9	179 (222)
	**0**	**1**	**2**	**3**	**4**	**5**	**6**	**7**	**8**	**9**

Desired Output

Supplementary Table 4 | Confusion matrix of the MNIST recognition test from the memristor-based RC system, using 88 memristors as the reservoir. The occurrences of the predicted outputs from the network are shown for each test case. The numbers in the parentheses show the total number of tests for each case.

Supplementary Note 1. WO_x memristor characterization

As with all memristor devices, a "pinched-hysteresis" behavior can be distinctively observed in the I-V characteristics in the WO_x memristor devices, as shown in Supplementary Figure 3. When a positive voltage is applied, the device conductance gradually increases (termed the write process) and when a negative voltage is applied the conductance gradually decreases (termed the erase process). Moreover, when multiple consecutive positive sweeps are applied, the device conductance continues to increase with each sweep, but also exhibits overlaps between the hysteresis loops, consistent with the short-term memory behavior discussed in Supplementary Reference [1].

The gradual conductance changes can be more clearly observed by pulse measurements, as shown in Supplementary Figure 4. Here 50 write pulses (+1.4 V, 100 µs) were applied to the device, followed by 50 erase pulses (-1.3 V, 100 µs). The device state was monitored by a small read pulse (0.5 V, 200 µs) after each write/erase pulse.

In another experiment, the device, from an initial state, was first subjected to five write pulses (1.4 V, 1 ms) then its state evolution was periodically monitored by applying small read pulses (0.4 V, 500 µs). As shown in Supplementary Figure 5, the conductance, indicated by the read current, was initially increased by write pulses then gradually decayed with time. This decay process can be fitted with an stretched exponential decay function discussed in Supplementary Reference [1], with a time constant ~50 ms.

The device characteristics can be explained by the redistribution of ions, here in the form of oxygen vacancies (V_Os), as has been discussed in Supplementary References [1–4].

Specifically, the memristor dynamics can be described by the following equations:

$$I = (1 - w)\alpha[1 - exp(-\beta V)] + w\gamma \sinh(\delta V) \qquad (1)$$

$$\frac{dw}{dt} = \lambda sinh(\eta V) - \frac{w}{\tau} \qquad (2)$$

where Supplementary Equation (1) is the I-V equation which includes a Schottky (the 1st term) corresponding to conduction in the V_O-poor region and a tunneling-like term (the 2nd term) corresponding to the V_O-rich region (Supplementary Reference [2]). The two conduction channels are in parallel and their relative weight is determined by the internal state variable *w*.

Supplementary Equation 2 is the dynamics equation which describes the change rate of the state variable w with respect to the applied voltage, including the drift effect under an applied electric field (the 1st term) and the spontaneous diffusion (the 2nd term). $\alpha, \beta, \gamma, \delta, \lambda, \eta$ are all positive-valued parameters determined by material properties. τ is the diffusion time constant.

In our memristor device, the short-term memory (STM) refers to the fact that some types of the memristor devices, including the ones used here can hold its conductance value for a short period of time (Supplementary Reference [1]). The WOx devices fabricated for this project were designed to have such a property, such that after being programed by voltage pulses that lead to an increase in device conductance, the device conductance will gradually decay to the original state (the state before the device is programmed). The memristor's short-term memory effect can be described by the time constant τ (Supplementary References [1,2]), and in the devices used in this study is ~ 50ms. As a result, when programming the device, the device state depends not only on the programming pulse itself, but also depends on whether other programming pulses have been applied within the immediate past within a period of around 50ms.

Supplementary Note 2. Memristor response to pulse streams

We selected 88 devices from the 32x32 memristor array for MNIST classification and 90 devices for the 2nd order nonlinear dynamic system analysis. Supplementary Figure 6 shows the actual locations of the 90 devices used for the 2nd order nonlinear dynamic system experiment. We selectd the 90 devices from the array in a way to avoid having adjacent devices in both row and column direction to minimize the write disturbance.

To verify the proper operation of the devices, the following experiment has been conducted. We send the same pulse streams to all the 90 devices individually and measure the device response. We found out that besides expected device to device variations, all devices can response to the input pulse sequence correctly and demonstrate similar current dynamics. The response to 4 different pulse squences from all 90 devices are shown in Supplementary Figure 7.

To verify that a unique input sequence will always lead to the same output, we performed a test where one memristor device in the reservoir is repeatedly tested, while the other devices remain unperturbed. In this case, since all other devices remain unchanged, the reservoir state can be represented by the resistance value of the device that is being stimulated. As seen in

Supplementary Figure 8, the same input sequence always leads to the same unique device response (and thus the overall reservoir state).

For the ten digits shown in Figure 2b that are represented by the 5×4 images, there are overall ten different possible pixel arrangements along each row direction, corresponding to ten different pulse stream patterns at the input to a memristor in the reservoir. We tested the memristor's response to these pulse streams, shown in Supplementary Figure 9. A different memristor state (as reflected by the read current after the pulse stream) was obtained for each pulse stream input, indicating that the memristor can separate those ten different pulse stream patterns.

To further verify that the memristor reservoir is sensitive to temporal reordering, in the following plot, we show experimental responses obtained from 4 different devices when subjected to 6 different input pulse streams. All input streams are variations having exactly two ONEs and two ZEROs. As can be clearly seen from Supplementary Figure 10, although the inputs all have the exact number of ZEROs and ONEs, the differences in the temporal ordering lead to distinct final current outputs from the device, due to the short-term memory effect of the device.

The uniqueness of the output for a given input is further verified by extensive simulations using a realistic device model (Supplementary Reference [2]). Supplementary Figure 11 confirms the uniqueness of the output for a more complex case with inputs consisting of 5 input pulses, where all possible input scenarios have been tested.

Supplementary Note 3. Simulation of handwritten digit recognition

Simulation of the memristor-based RC system was conducted using Matlab 2016b. Following the experimental procedures discussed in the main text, the original MNIST data was preprocessed, and converted into pulse streams to be fed to the memristor-based reservoir. The memristor response was simulated using the memristor model in Supplementary Equations 1-2, with parameters given in Supplementary Table 1.

The memristor was initialized to w=0.1 before each pulse stream, corresponding to the resting state of the device. The device evolution during the pulse stream can be separated into two regimes:

1) when a write pulse is applied, the state variable w will be changed by:

$$\Delta w = R(w) * t_{pulse} * \lambda * sinh(\eta * V_{pulse}) \quad (3)$$

where t_{pulse} and V_{pulse} are the width and amplitude of the write pulse, respectively. The decay effect (the 2nd term in Supplementary Equation (2)) is neglected during the write pulse. $R(w)$ is a window function[2]) to ensure the state variable w will not exceed its upper limit (w=1) and is chosen to be:

$$R(w) = 1 - \frac{exp(w*3)}{exp(w_{Max}*3)}, \text{ with } w_{Max} = 1 \quad (4)$$

2) during the period when a write pulse is not applied, the state variable w will decay as:

$$\Delta w = (w_0 - w_{Min}) * \left(1 - exp\left(-\frac{\Delta t}{\tau}\right)\right), \text{ with } w_{Min} = 0.1 \quad (5)$$

where Δt is the length of this period and w_0 is the value of w at the beginning of the period. This effect can be easily derived from the integral of Supplementary Equation 2 by letting V=0.

After a pulse stream is fed into the memristor, a read pulse is applied and the corresponding read current is calculated using Supplementary Equation 1. The reservoir state is represented by a vector consisting of the read currents of all the memristors in the reservoir. For the case of a 24×24 image with 4 sections and 3 input rates, the vector's length is 288.

Supplementary Table 2 shows the write pulse, read pulse and input rates used in simulation, to be made close to the conditions used in the experiments.

During simulation, the 60,000 training samples from the MNIST dataset are fed to the memristor-based reservoir. The reservoir states are then obtained and used to train the readout function by logistic regression discussed earlier. After training the readout function, 14,000 test samples not in the training set are used to test the system's ability to recognize the handwritten digits. Supplementary Table 3 summarizes results obtained from the simulation and experimental studies for different network configurations.

Supplementary Table 4 shows the confusion matrix listing the MNIST recognition outputs from the experimental study, using 88 memristors as the reservoir.

Supplement Note 4. Benchmarking of the memristor based reservoir computing system

To verify the function of the memristor reservoir layer, we added a hidden layer to the FF network case with connectivity patterns that reflect the priors of memristor-based RC constraints. In the memristor reservoir case, each memristor device transforms multiple inputs into a single output (Supplementary Figure 12a) to the readout layer. To obtain a network with the same connectivity pattern, we added a conventional hidden layer, i.e, a commonly used cubic interpolation downsampling function, that similarly reduces multiple consecutive pixels into a single output to the readout layer (Supplementary Figure 12b). With this approach, we can compare the two systems at equivalent network sizes and connectivity patterns.

Following our experimental procedure, each row in the original (22×20) MNIST image is divided into sections of m consecutive pixels (where m = 20, 10, 5, 4, 2) to ensure the sections do not overflow to the next row), and both the reservoir layer and the downsampling function can convert the consecutive pixel data in a section into a single output, i.e. with a downsampling ratio of m:1. This analysis thus allows us to directly compare the functions of the reservoir layer with that of a conventional hidden layer, i.e. a downsampling layer that leads to the same connectivity patterns as the RC system case. Results obtained from both networks at different sizes (Supplementary Figure 13) show that for a given readout network size, the RC system generally outperforms the traditional system with a downsampling layer. The improvement is more significant at smaller network sizes. In other words, besides compressing the input (which is the sole purpose of the downsampling function), the memristor reservoir layer is able to better utilize the pulse patterns in the input and map the different input patterns to an output space that offers improved classification results through the readout layer. At larger network sizes, e.g. with readout network size of 22×5×10, corresponding to a downsampling ratio of 4:1, the performance of both systems saturates and approaches that of the uncompressed input case, since the inputs of 4 consecutive pixels become sufficiently similar and downsampling in this case leads to little information loss.

This analysis shows that the memristor reservoir indeed offers more functionality than a conventional nonlinear downsampling layer, and leads to improved network performance, particularly at small network sizes.

Supplementary Note 5. Comparison of the 2nd order nonlinear task performance with a conventional linear network

It worth mentioning that all the preprocessing and training operations used in the 2nd order nonlinear dynamic task are based on linear transform. As a result, the nonlinear transformation required by the task has to originate from the intrinsic nonlinear physics of the memristor device.

To highlight the computing capacity provided by the memristor reservoir, we compared the RC system performance against a linear network of the same size. We replaced the memristor reservoir layer with a linear hidden layer, which generates 90 randomly linearly scaled signals of the original input u(k), with scaling factors:

$$\mathrm{x(k)} = 2 * \mathrm{rand}(0{,}1) * \mathrm{u(k)} \qquad (6)$$

Where the x(k) is scaled and from the original input signal u(k), similar to a current value through a linear resistor. In this case, there is no longer any nonlinear transform provided by the reservoir.

We then repeated the signal reconstruction experiments in Figures. 5c and 5e using the linear network with the same training and test data sets and the same training procedure. The results (Supplementary Figure 14) show that the linear network is not able to solve the dynamic nonlinear problem, and exhibits large errors of 2.23×10^{-1} for the training set and 1.67×10^{-1} for the test set.

We also calculated the output NMSE for the linear network and the memristor RC system vs. the readout network size (which equals the number of devices *n* in the reservoir layer since the readout layer is an $n\times1$ network). Here results from the memristor-based RC system were obtained experimentally using the test board, while results from the linear network were obtained from software. As can be observed from Supplementary Figure 15, the memristor RC system significantly outperforms the linear network having the same size, when solving this dynamic nonlinear task. Additionally, the performance of the memristor RC system is generally improved when using multiple memristor devices in each group, since the inherent device variations increases the reservoir output dimension and thus help improve reservoir state separation.

Supplementary References

1. Chang, T., Jo, S. H. & Lu, W. Short-term memory to long-term memory transition in a nanoscale memristor. *ACS Nano* **5,** 7669–7676 (2011).

2. Chang, T. *et al.* Synaptic behaviors and modeling of a metal oxide memristive device. *Appl. Phys. A* **102,** 857–863 (2011).

3. Strachan, J. P. *et al.* State dynamics and modeling of tantalum oxide memristors. *IEEE Trans. Electron Devices* **60,** 2194–2202 (2013).

4. Strukov, D. B. & Williams, R. S. Exponential ionic drift: Fast switching and low volatility of thin-film memristors. *Appl. Phys. A* **94,** 515–519 (2009).
